# Controversies in the management of endophthalmitis: a 5-year retrospective cohort study

**DOI:** 10.1186/s12348-025-00468-8

**Published:** 2025-03-17

**Authors:** Ohisa Harley, Yufilia Suci Amelia, Elsa Gustianty, Nanny N. M. Soetedjo, Arief S. Kartasasmita

**Affiliations:** 1 Doctoral Program in Medical Sciences, Faculty of Medicine, Padjadjaran University, Ir. Soekarno Street Km 21, Jatinangor, Bandung, 45363 West Java Indonesia; 2 Netra Eye Clinic Centre, Sumatera Street No. 46-68, Bandung, 40114 West Java Indonesia; 3 Departement of Ophthalmology, Faculty of Medicine, Padjadjaran University, Ir. Soekarno Street Km 21, Jatinangor, Bandung, 45363 West Java Indonesia; 4 Departement of Endocrinology and Internal Medicine, Faculty of Medicine, Padjadjaran University, Ir. Soekarno Street Km 21, Jatinangor, Bandung, 45363 West Java Indonesia; 5 Cicendo Hospital National Eye Center, Cicendo Street No.4, Babakan Ciamis, Bandung, 40117 West Java Indonesia

**Keywords:** Vitrectomy, Endophthalmitis, Prognostic factor, Intravitreal antibiotic, Steroid

## Abstract

**Background and purpose:**

Post-operative endophthalmitis has a risk of vision loss if the treatment is delayed. Managing endophthalmitis based on visual outcome has become controversial. This study primarily aimed to evaluate the effectiveness of early pars plana vitrectomy (PPV) combined with intravitreal antibiotics in improving visual acuity and reducing complications in patients with post-operative endophthalmitis. Secondary objectives included identifying prognostic factors influencing visual outcomes after PPV, assessing the effectiveness of different intravitreal antibiotic regimens on visual recovery, and evaluating the role of steroid as adjunctive therapy in influencing visual outcome and controlling inflammation.

**Methods:**

A 5-year retrospective cohort study was conducted, reviewing medical records of patients diagnosed with post-operative endophthalmitis between 2019 and 2023. Data collected included patient demographics, medical and surgical history, culture results, treatments administered, baseline best-corrected visual acuity (BCVA), and BCVA outcomes within three months after vitrectomy.

**Results:**

40 eyes of 40 patients with acute post-operative endophthalmitis underwent early PPV followed by intravitreal antibiotics. Median logMAR BCVA improved from 2.0 at presentation to 0.4 three months post-vitrectomy (*p* < 0.05), with a mean final logMAR BCVA of 0.94 ± 1.13. No significant difference was observed in visual outcomes or complication rates between patients treated with intravitreal vancomycin and ceftazidime versus moxifloxacin monotherapy. Univariate analysis identified high intraocular pressure (*p* = 0.004, β = 2.42), hypopyon (*p* = 0.01, β = 1.79), and a history of surgery more than seven days prior (*p* = 0.032, β = 1.74) as significant predictive of visual outcomes. Multivariate analysis confirmed intraocular pressure (*p* = 0.008, β = 2.55) and surgical history (*p* = 0.045, β = 1.84) as independent predictors. Baseline BCVA, fibrin, retinal findings, and symptom onset were not significantly associated with outcomes. Neither antibiotics regimen nor steroid use significantly influenced treatment results.

**Conclusion:**

This study supports performing early PPV combined with intravitreal antibiotics as an effective primary treatment to improve visual outcomes in post-operative endophthalmitis. Negative prognostic factors included hypopyon, elevated intraocular pressure, and a surgical history of more than seven days. Management should prioritize clinical signs over microbiological culture results to prevent delays in treatment.

## Introduction

Endophthalmitis represents a critical intraocular inflammatory condition, most frequently arising as a postoperative complication following intraocular surgery. The incidence of this condition varies globally, with reported rates ranging from 0.03–0.7% [1, 2]. Despite technically successful and uneventful surgeries, endophthalmitis remains a potential and serious postoperative complication [3, 4] The condition poses a significant threat to vision, with outcomes depending heavily on the pathogen, the severity of inflammation, and the speed and accuracy of treatment. Delays in treatment can lead to a substantial risk of permanent blindness [1, 3]. 

Controversies persist regarding the optimal management of endophthalmitis, encompassing discussions on the timing and technique of vitrectomy, antibiotic selection, and the use of adjunctive steroid therapy [5]. Although some studies have shed light on the advantages and drawbacks of these various approaches, the recommendations established by the Endophthalmitis Vitrectomy Study (EVS) may not fully correspond to contemporary clinical practices [6–9]. This study seeks to critically evaluate the current controversies surrounding endophthalmitis management and to elucidate the prognostic factors that affect visual outcomes in patients with postsurgical endophthalmitis.

## Materials and methods

This retrospective cohort study analyzed medical records of acute post-operative endophthalmitis managed at Netra Eye Clinic, West Java, Indonesia. Cases were identified using the ICD-10 code for endophthalmitis, and data were collected from January 2019 to January 2024. This study was performed according to the tenets of Declaration of Helsinki. Ethical clearance was obtained from Padjadjaran University ethical committee.

### Criteria selections

The inclusion criteria were: (1) acute post-surgery endophthalmitis within 4 weeks post-surgery (2) patients with or without culture examination results. The exclusion criteria include (1) other causes of endophthalmitis, (2) endogenous and post-traumatic cases, (3) patients who did not receive PPV, and (4) incomplete data or lost to follow-up.

We collected demographic data, including age and gender, alongside ocular and medical history, surgical details, clinical presentation, and treatment outcomes. A detailed history of prior ocular surgery was recorded, including the type of surgery and the duration from the procedure to presentation at the clinic. The treatment was done at the same day of the presentation. The onset from previous surgery to diagnosis of endophthalmitis was defined as the time interval between the last ocular surgery and the treatment. The duration of manifestation was defined as the interval from the first reported day of symptoms, as documented through anamnesis, to the treatment. We divided into three categories: ≤ 3 days, 4–7 days, and > 7 days.

BCVA at baseline and three months post-treatment was assessed using a Snellen chart. All VA measurements were converted to logMAR for statistical analysis. VA calssifications included: ≤ logMAR 0.4, logMAR 0.7 to logMAR 1.7, and > logMAR 1.7. VA values for count finger (CF), hand movement (HM), light perception (LP), and no light perception (NLP) were assigned with values of 2.0 logMAR, 2.3 logMAR, 3.0 logMAR, 4.0 logMAR units respectively. Intraocular pressure (IOP) was measured using Non-Contact Tonometry (NCT) at presentation. Cases with IOP > 21 mmHg were documented as elevated.

Clinical signs of inflammation, including the presence of fibrinous membrane and hypopyon, were carefully documented. Hypopyon measurements were categorized as ≥ 1 mm or < 1 mm. Indirect funduscopy was used to assess the vitreous haze, retinal vascular sheathing, and retinal exudates. B-scan USG results were reviewed for vitreous opacity and the presence of a double-layer sign. These findings were categorized into clinically positive or negative signs. Clinically positive retinal signs include (1) double-layered vitreous opacity observed in B-scan USG, (2) exudates or vascular sheathing in the retina, or (3) vitreous suppuration. Cases without these features were classified as negative. Microbiological analysis, including Gram/KOH staining and culture, was conducted on vitreous tap samples when available.

Variable analyzed were age, gender, type of previous surgery, onset from previous surgery, duration of manifestation, clinical features (fibrin and hypopyon), intraocular pressure (IOP), baseline BCVA, BCVA at three months, treatment regimens (types and numbers), complications, and microbiological findings. Retinal examination feature was analyzed categorically as clinically positive or negative based on the predefined criteria.

### Treatment regimen

In our retrospective study, all cases of post-operative acute endophthalmitis underwent early PPV followed by intravitreal antibiotic injection (IVAB) within 24 h of diagnosis. The procedure was performed by one of the retina surgeons (AF, EN, and OH) under peribulbar anesthesia using 23-gauge instruments. A vitreous tap was conducted prior to initiating cannula infusion. When necessary, anterior chamber washout and/or membrane removal around the pupil were performed to enhance visualization. Core vitrectomy was carried out with an attempt to induce posterior vitreous detachment when feasible, although this step was not mandatory. Efforts were made to minimize intraocular manipulation.

After completing the core vitrectomy, intravitreal antibiotics were administered. From data, there are two intravitreal antibiotic regimens were used: (1) a combination of vancomycin 1 mg/0.1 ml and ceftazidime 2.25 mg/0.1 ml, or (2) moxifloxacin 0.5 mg/0.1 ml. In some cases, corticosteroid was administered, using intravitreal injection of dexamethasone 0.4 mg/ 0.1 ml and/or subtenon injection of triamcinolone acetonide 20 mg/0.5 ml. Systemic antibiotics were given in selected cases based on clinical judgment.

### Study group, outcome measures and statistical analysis

This study primarily aimed to evaluate the effectiveness of early PPV combined with intravitreal antibiotics in improving visual acuity and reducing complications in patients with post-operative endophthalmitis. Secondary objectives included identifying prognostic factors influencing visual outcomes after PPV, assessing the effectiveness of different intravitreal antibiotic regimens on visual recovery, and evaluating the role of adjunctive therapies in influencing visual outcome and controlling inflammation.

To evaluate the effectivity of management, we divided patients into two groups for analysis based on final BCVA after PPV: (1) Good outcome, which final BCVA at three months was ≤ logMAR 0.4, and (2) Poor outcome, which final BCVA at three months was > logMAR 0.4.

Continuous variables were expressed as means ± standard deviations or median (range) when data were not normally distributed. The Saphiro-Wilk test was used to assess the data normality, and categorical data were summarized as frequencies and percentages. Wilcoxon signed ranked test were used to evaluate the change of BCVA from baseline. Chi-Square and Fisher’s exact were used to evaluate the differences in categorical variables, e.g. proportion of good and poor outcomes. Univariate and multivariate linear regression analyses were used to assess the relationship between prognostic factors and outcome of PPV. Also, all probability values (*p-*value) were deemed statistically significant at a level of < 0.05. Statistical analyses were performed with SPSS, version 23.0 SPSS, Inc, Chicago, IL, USA.

## Results

### Baseline characteristics

**Fig. 1:**
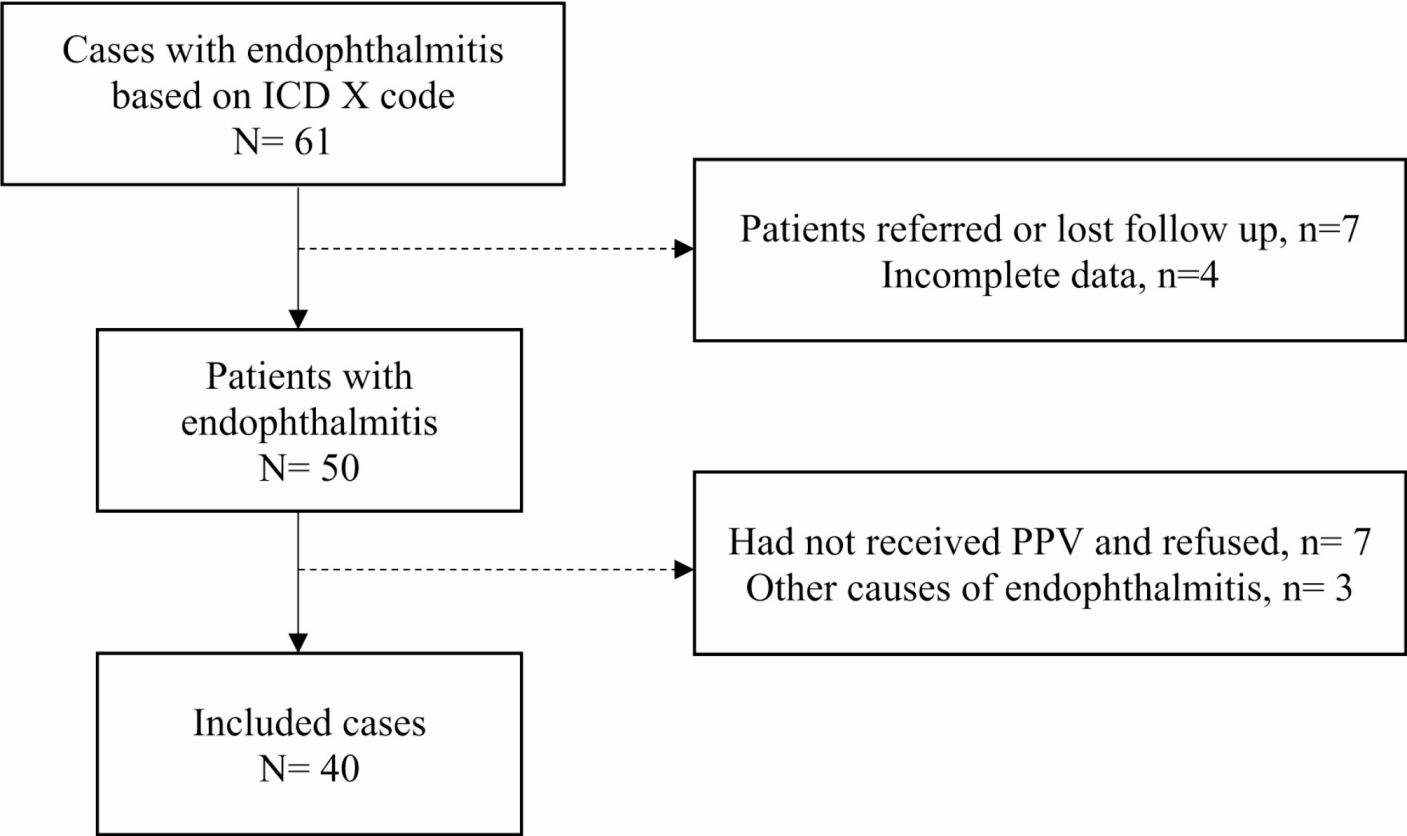
Criteria of selection

A total of 40 eyes from 40 patients with acute post-operative endophthalmitis met the inclusion criteria (Fig. 1). The mean age of patients was 62.52±10.23 years (range 28–79 years). There were 13 males (32.5%) and 27 females (67.5%). Systemic comorbidities observed among the patient included hypertension, diabetes mellitus, and heart disease. Most cases were considered uneventful surgeries. However, there were three cases of cataract surgeries associated with intraoperative complications: two cases (9.7%) were complicated by posterior capsular rupture (PCR), and one case had vitreous prolapse (3.7%).

### Presenting clinical features

In this study, 15 cases with baseline BCVA > logMAR 1.7 and 8 cases with baseline BCVA logMAR 0.7 to logMAR 1.7 had good outcomes after undergoing early PPV with IVAB. The mean of logMAR BCVA on presentation was 1.91±0.74. Patients presented at a mean time of 6.85±6.95 days post-operative with eye pain, redness, and blurring of vision. The mean duration of manifestation before presentation was 3.07 ± 4.92 days.

Significant hypopyon ≥ 1 mm, fibrin was found at 37.5% and 75% in our study, respectively. A history of secondary glaucoma related to eye disorders was observed in 17.5% of cases. However, on the day of the presentations, we found a high IOP > 21 mmHg in 22.5% of cases. The mean IOP was 22.20±15.12 mmHg. Clinically positive retina features observed were vitreous suppurative or opacity in 18 cases (45%), retinal exudates in 16 cases (40%), and vascular sheathing in 11 cases (27.5%). There were no significant differences between gender, baseline VA, fibrin, retina sign, comorbidity, type of surgery, and duration of manifestation. The detailed characteristics are shown in Tables 1 and 2.

**Table 1:** Baseline characteristics of patients

Characteristics		Outcome PPV		*p*-value
		Good *n* = 27	Poor *n* = 13	
Age (mean ± SD)		63.96 ± 8.63	59 ± 12.74	0.2
Gender				
	Male	9 (34.6%)	4 (28.6%)	
	Female	17 (65.4%)	10 (71.4%)	1.0
Type of previous surgery				
	Phacoemulsification	25 (96.2%)	12 (85.7%)	
	SICE	0%	1 (7.1%)	
	Intravitreal triamcinolone	1 (3.8%)	0%	
	Anti-VEGF intravitreal injection	0%	1 (7.1%)	0.27
History of ocular disease				
	Secondary Glaucoma	3 (11.5%)	4 (28.6%)	0.214
Comorbidities				
	Diabetes Melitus	3 (11.5%)	3 (21.4%)	0.64
	Hypertension	10 (38.5%)	3 (21.4%)	0.316
	Heart Disease	1 (3.8%)	0	1.0

*Significant for *p*-value < 0.05

BCVA = Best corrected visual acuity, IOP = intraocular pressure, SICE = Small incision cataract extraction, anti-VEGF = anti-vascular endothelial growth factor

**Table 2:** Presenting clinical manifestation between two groups

Characteristics		Outcome PPV		*p*-value
		Good *n* = 27	Poor *n* = 13	
BCVA at presentation				
	> logMAR 1.7	15 (57.7%)	11 (78.6%)	
	logMAR 1.7 to 0.7	8 (30.8%)	3 (21.4%)	
	≤ logMAR 0.4	3 (11.5%)	0 (0%)	0.19
IOP				
	> 21	2 (7.7%)	7 (50%)	
	≤ 21 mmHg	24 (92.3%)	7 (50%)	0.004*
Hypopyon				
	≥ 1 mm	6 (23.1%)	9 (64.3%)	
	< 1 mm	20 (76.9%)	5 (35.7%)	0.01*
Fibrinous Membrane around Pupil				
	Yes	18 (30.8%)	12 (85.7%)	
	No	8 (30.8%)	2 (14.3%)	0.4
Retinal Examination Results				
	Clinically Positive	23 (88.5%)	13 (92.9%)	
	Negative	3 (11.5%)	1 (7.1%)	1.0
Onset from previous surgery to treatment				
	> 7 days	3 (11.5%)	6 (42.9%)	
	≤ 7 days	23 (88.5%)	8 (57.1%)	0.044*
Duration of manifestation				
	> 7 days	1 (3.7%)	1 (7.1%)	
	4–7 days	6 (25.9%)	3 (21.4%)	
	≤ 3 days	19 (70.4%)	10 (71.4%)	0.85

*Significant for *p*-value < 0.05

IOP = intraocular pressure, BCVA = Best corrected visual acuity

### Treatments and complications

PPV was performed along with IVAB for all patients without the use of endo-tamponade. All cases received topical antibiotics and prednisolone postoperatively. Based on the clinical features and severity of endophthalmitis, some cases were also treated with intravitreal dexamethasone, triamcinolone acetonide, and systemic antibiotics. The treatment regimen involving intravitreal dexamethasone, subtenon triamcinolone acetonide and systemic antibiotics showed no statistically significance in visual outcomes across groups. Details of treatment regimens are shown in Table 3. The mean duration of systemic antibiotics was 4.68±1.99 days (3–10 days). Intravenous antibiotics administered included carbapenems, aminoglycosides, and third-generation cephalosporins. Oral antibiotics used included fluoroquinolones, sulfonamides, and penicillins. For suspected fungal infections, triazole antifungal agents were prescribed. The choice of systemic antibiotics was guided by the clinical judgment of vitreoretinal specialist while awaiting microbiological culture results.

Intraoperatively, no complications were recorded for any case. However, four cases developed post-vitrectomy complications: two cases of retinal detachment, one case of vitreous hemorrhage, and one case of recurrent retinal detachment. Secondary vitrectomy was performed to address these complications.

**Table 3:** Comparison of treatment between two groups

Treatment		Outcome PPV		*p*-value
		Good *n* = 27	Poor *n* = 13	
PPV				
	No	0	0	
	Yes	27 (67.5%)	13 (32.5%)	
Intravitreal Antibiotics				
	No	0	0	
	Yes	27 (67.5%)	13 (32.5%)	
Intravitreal antibiotics type				
	Moxifloxacin	14 (53.8%)	7 (50%)	
	Vancomycin and ceftazidime	12 (46.2%)	7 (50%)	0.81
Intravitreal Dexamethasone				
	No	17 (65.4%)	12 (85.7%)	
	Yes	9 (34.6%)	2 (14.3%)	0.27
Subtenon Triamcinolone Acetonide				
	No	16 (61.5%)	9 (64.3%)	
	Yes	10 (38.5%)	5 (35.7%)	1.0
Systemic antibiotics				
	Not received	13 (50%)	9 (64.3%)	
	Intravenous	7 (26.9%)	5 (35.7%)	1.0
	Oral	6 (23.1%)	0 (0%)	0.13

*Significant for *p*-value < 0.05

### Visual outcomes and prognostic factors

The overall median logMAR BCVA improved significantly, decreasing from a baseline of 2.0 logMAR unit to 0.4 logMAR unit at three months post-treatment (*p-value* < 0.05, Z= -4.526). The mean final logMAR BCVA was 0.94±1.13 logMAR unit. Four cases (10%) showed no improvement in BCVA from baseline to the final post-treatment measurement following PPV.

Statistical analysis revealed that elevated IOP, the presence of hypopyon, and the time interval between previous surgery and treatment were significantly different between the good and poor outcome groups (*p* = 0.004; *p* = 0.01; *p* = 0.044 respectively) (shown in Table 2). No significant differences were observed for the presence of fibrin, retinal clinical features, duration of manifestation, or systemic comorbidities between the two outcome groups (*p* > 0.05). In multivariate regression analysis, only the increase of IOP and the time interval from previous surgery to treatment were identified as statistically significant prognostic factors. The IOP showed an odd ratio (OR) of 12.89 (95%CI (1.93–85.92), *p* = 0.008, β **=** 2.55), while the interval previous surgery to treatment has an OR of 6.32 (95%CI (1.04–38.46), *p* = 0.045, β **=** 1.84). These findings indicate a positive β correlation, demonstrating that the higher IOP and a longer time interval from previous surgery to treatment were associated with worse final BCVA outcomes after PPV (Table 4).

**Table 4:** Univariate and Multi-regression analysis prognostic factors for PPV outcome

Characteristics / Clinical feature	Univariate regression analysis			Multivariate regression analysis		
	OR	*p*-value	β coefficient	OR	*p*-value	β coefficient
Hypopion	6.00 (1.44–24.91)	0.01*	1.79	3.77 (0.66–21.6)	0.135	1.32
IOP	12.00 (2.01–71.35)	0.004*	2.42	12.89 (1.93–85.92)	0.008*	2.55
Onset from previous surgery to treatment	5.75 (1.15–28.55)	0.032*	1.74	6.32 (1.04–38.46)	0.045*	1.84

*Significant for *p*-value < 0.05

### Bacterial characteristics

Vitreous tap samples from 27 out of 40 eyes were examined using Gram staining and KOH preparation, with the detailed results presented in Table 5. Meanwhile, bacterial cultures yielded positive results in only two cases, identifying Pseudomonas fluorescens and Seratia marcescens as the causative microorganisms.

**Table 5:** Gram/KOH results

Vitreous Tap (*n* = 27)	Outcome PPV	
	Good	Poor
Gram Positive	4 (23.5%)	3 (33.3)
Gram negative	2 (11.8%)	0%
Combined (gram positive and negative)	1 (5.9%)	1 (11.1%)
Fungal	1 (5.9%)	0%

## Discussion

This retrospective cohort study offers several critical insights into the management of postoperative endophthalmitis. The findings highlight the importance of early intervention, specifically the implementation of PPV combined with intravitreal antibiotic injection within 24 h of clinical presentation. This proactive approach was employed across all cases, including those with relatively preserved baseline visual acuity (< 0.7 LogMAR), emphasizing a strategy designed to minimize the risk of vision loss. Similar findings in other studies also underscore the benefits of early vitrectomy in acute endophthalmitis [7–12]. 

The significant findings of this retrospective cohort study are as follows. First, the uniform application of early PPV combined with intravitreal antibiotics demonstrates a robust treatment protocol, irrespective of initial visual acuity. In this study, broad-spectrum antibiotics were administered without alteration based on subsequent culture results. This standardized approach may reflect a precautionary principle that prioritizes immediate antimicrobial coverage in the absence of definitive microbiological evidence.

Second, the study identified several negative predictive factors for visual outcomes, including the presence of hypopyon, elevated intraocular pressure (IOP), and a prolonged interval (> 7 days) between the inciting surgical procedure and the onset of endophthalmitis. These variables likely indicate greater disease severity or microbial virulence. The presence of hypopyon, in particular, may represent a higher microbial load or a more virulent pathogen, both contributing to worse outcomes.

The management of endophthalmitis revolves around three principal objectives: (1) the eradication or control of infection, primarily through surgical intervention (vitrectomy) and antimicrobial therapy; (2) the suppression of inflammation, which is crucial in preventing irreversible damage to ocular structures, particularly the neurosensory retina; and (3) supportive care, including the management of intraocular pressure and the use of adjunctive therapies. Options are available, and decisions are usually made based on the clinical feature and severity level of endophthalmitis.

EVS guidelines recommend treatment based on BCVA: patients with BCVA > logMAR 2.30 were treated with IVAB alone, while those with BCVA ≤ logMAR 2.30 underwent PPV combined with IVAB. However, cases with BCVA ≤ logMAR 2.30 are often in advanced stages, and full visual recovery is unlikely even after the resolution of endophthalmitis. This may be due to the spread of intraocular inflammation causing damage to the retina and optic nerve, leading to macular ischemia and optic disc atrophy. With advancements in surgical instruments, PPV nowadays may serve as an early treatment option for acute post-surgical endophthalmitis, offering greater benefits by immediately eradicating bacteria and preventing further retinal damage. Despite its effectiveness, like any surgery, PPV has its surgical risks of complications which need to be further studied.

The 2013 guidelines from the European Society of Cataract and Refractive Surgeons (ESCRS) advocate for complete vitrectomy as the gold standard in the management of post-cataract surgery endophthalmitis [13]. This recommendation is based on the premise that complete vitrectomy facilitates the removal of infectious agents and inflammatory mediators, thereby reducing the risk of persistent infection and improving visual outcomes. Subsequent cohort studies have reinforced the efficacy of this approach, particularly when implemented as an initial treatment strategy [12, 14]. In our study, the majority of cases (92.5%) occurred following cataract surgery, and early PPV was associated with favorable outcomes in a significant proportion of cases. However, the severity of inflammation often precluded the performance of complete PPV, necessitating a more conservative approach involving core vitrectomy, with or without the induction of posterior vitreous detachment. Despite this limitation, more than 67% of cases achieved a final visual acuity of better than 2.3 LogMAR, highlighting the potential benefits of early surgical intervention even when complete vitrectomy is not feasible.

The choice of antibiotic regimen before obtaining the results of vitreous tap cultures remains a subject of ongoing debate [15]. This study found no significant difference in visual outcomes or complication rates between patients treated with the traditional combination of intravitreal vancomycin and ceftazidime versus those treated with moxifloxacin monotherapy. The combination of vancomycin and ceftazidime, as recommended by the EVS, remains a widely accepted standard, offering broad-spectrum coverage against both gram-positive and gram-negative bacteria [16–18]. Moxifloxacin, a fourth-generation fluoroquinolone, has gained popularity due to its broad-spectrum activity and its ability to inhibit DNA gyrase, an enzyme crucial for bacterial DNA replication. This mechanism of action may confer an advantage in reducing the development of bacterial resistance, particularly when compared to antibiotics that target cell wall synthesis, such as vancomycin and ceftazidime. The broad antimicrobial coverage provided by moxifloxacin makes it a suitable option for empirical therapy in cases of endophthalmitis, especially when the causative organism is unknown. Comparative studies have shown that the use of high-dose moxifloxacin alone is as effective as combination therapy with vancomycin and ceftazidime, with no significant differences in clinical outcomes [19]. 

The use of adjunctive systemic antibiotics in the treatment of endophthalmitis also remains controversial. While some studies support their use in severe cases of acute, purulent postoperative endophthalmitis [20], our study did not find a statistically significant association between the administration of systemic antibiotics and improved visual outcomes. However, this result should be interpreted with caution, as there may be a selection bias; systemic antibiotics were more likely to be administered to patients presenting with more severe inflammation.

The effectiveness of systemic antibiotics is influenced by their ability to penetrate the blood-retinal barrier (BRB), a key factor in the treatment of intraocular infections. Antibiotics such as moxifloxacin and imipenem have been shown to cross the BRB more effectively than others, such as vancomycin and amikacin. However, the inflammatory process inherent to endophthalmitis may disrupt the integrity of the BRB, thereby enhancing the penetration of systemic agents into the eye [21]. Therefore, the administration of systemic antibiotics may be considered for severe endophthalmitis by surgeon judgment.

Intravitreal steroids along with antimicrobials for endophthalmitis management have also been marred with controversy for decades. The rationale behind steroid use lies in their ability to suppress inflammation, thereby potentially preventing irreversible damage to the neurosensory retina [22]. This is particularly relevant in cases where the inflammatory response is driven not only by the infecting organism but also by the host’s immune response. However, caution is required in fungal endophthalmitis cases, as steroids can worsen the outcomes.

Our study did not find a statistically significant association between intravitreal dexamethasone administration and final visual outcomes. This finding aligns with other studies that have shown no significant long-term benefits of intravitreal steroids in the treatment of endophthalmitis [23–26]. In our cohort, there was a tendency to administer intravitreal dexamethasone combined with triamcinolone sub-tenon injections in the most severe cases—those characterized by worse presenting visual acuity, more pronounced inflammation (e.g., chemosis, hypopyon), and challenges in visualizing the posterior pole. These cases, given their severity, were likely to have poorer outcomes regardless of treatment. The half-life of intravitreal dexamethasone is approximately 5.5 h, but when combined with triamcinolone sub-tenon injection, the duration of steroid action may be extended to 1–2 weeks, offering prolonged anti-inflammatory effects. Additionally, systemic steroids, though not found to be significantly beneficial in this study, have been reported in some cases to not worsen long-term outcomes when used in conjunction with intravitreal steroids [24, 26–28]. 

This cohort indicates that factors contributing to poor VA outcomes included hypopyon, elevated IOP, and time interval between surgery and treatment of more than 7 days. Multivariate regression analysis indicated that poor final visual acuity was significantly associated with the time interval between the previous surgery and vitrectomy (*P* = 0.016; ß coefficient 2.52; OR 25.31 95%CI (1.59–98.81)) as well as IOP (*P* = 0.003; ß coefficient: 3.23; OR 25.31 95%CI (2.98-214.97)). The onset of endophthalmitis from surgery was significantly associated with visual outcome [29, 30]. These findings highlight the importance of clinicians being aware of timely intervention where post-operative endophthalmitis involving virulent microorganisms poses higher risk of poor visual outcome with time. Additionally, our study found that traditional markers of severe inflammation, such as poor baseline visual acuity, dense vitreous haze, and positive retinal signs, did not necessarily predict poor outcomes if early PPV was performed. This finding supports the argument that early surgical intervention, coupled with appropriate antimicrobial therapy, can mitigate the adverse effects of severe inflammation and improve the likelihood of visual recovery. Furthermore, our data showed that the mean duration of manifestation was three days or longer. This finding reflects a low level of patient awareness regarding the urgency of seeking clinical evaluation. These results highlight the critical need to improve patient education on recognizing the symptoms of endophthalmitis and the importance of prompt medical attention. Early recognition of symptoms facilitates timely diagnosis and treatment, which can help prevent further damage.

This study has several strengths. It represents a 5-year cohort analysis, providing a substantial dataset in the context of the relatively low incidence of endophthalmitis. The uniformity of the treatment approach—early core vitrectomy combined with intravitreal antibiotics and, in some cases, adjunctive steroid therapy—adds to the consistency and reliability of the findings. Nearly two-thirds of the cases in this study resulted in favourable visual outcomes, supporting the efficacy of this approach.

This study has several limitations. The sample size may be inadequate to provide conclusive results, and the reliance on self-reported symptom onset introduces potential recall and response bias. The absence of culture examinations in some cases limits the ability to correlate microbial data with clinical outcomes. Moreover, the retrospective design and a relatively short follow-up period of three months may not adequately reflect long-term outcomes or capture late-onset complications. Future prospective, randomized controlled trials are needed to advance the management of endophthalmitis strategies, with a focus on optimizing treatment protocols to minimize the risk of permanent visual impairment.

## Conclusion

The findings of this study underscore the importance of early core PPV combined with IVAB injection as a primary treatment strategy for post-operative endophthalmitis. This approach significantly increases the likelihood of achieving favourable visual outcomes, even in cases presenting with severe inflammation. Both vancomycin-ceftazidime and moxifloxacin demonstrated comparable efficacy in managing infections, and the use of systemic antibiotics did not significantly impact visual outcomes. The study also identified hypopyon, an elevated IOP, and the surgical history of more than 7 days as negative predictive factors for visual prognosis. The management of post-operative endophthalmitis should be guided by clinical signs and disease severity rather than relying solely on microbiological culture results. Furthermore, although performing PPV has been shown to be an effective intervention, it is associated with potential complications that require further investigation.

## Data Availability

No datasets were generated or analysed during the current study.

## Abbreviations

BCVA: Best corrected visual acuity
BRB: Blood-retinal barrier
CF: Count finger
ESCRS: European Society of Cataract and Refractive Surgeons
EVS: Endophthalmitis Vitrectomy Study
HM: Hand movement
ICD: International Classification of Diseases
IOP: Intraocular pressure
IVAB: Intravitreal antibiotic injection
LP: Light perception
NCT: Non-Contact Tonometry
NLP: No light perception
PCR: Posterior capsular rupture
PPV: Pars plana vitrectomy
SICE: Small incision cataract extraction
